# *In vivo* reprogramming reactive glia into iPSCs to produce new neurons in the cortex following traumatic brain injury

**DOI:** 10.1038/srep22490

**Published:** 2016-03-09

**Authors:** Xiang Gao, Xiaoting Wang, Wenhui Xiong, Jinhui Chen

**Affiliations:** 1 Spinal Cord and Brain Injury Research Group, Stark Neuroscience Research Institute, Department of Neurosurgery, Indiana University, 320 W 15th Street, Indianapolis, IN 46202.

## Abstract

Traumatic brain injury (TBI) results in a significant amount of cell death in the brain. Unfortunately, the adult mammalian brain possesses little regenerative potential following injury and little can be done to reverse the initial brain damage caused by trauma. Reprogramming adult cells to generate induced pluripotent stem cell (iPSCs) has opened new therapeutic opportunities to generate neurons in a non-neurogenic regions in the cortex. In this study we showed that retroviral mediated expression of four transcription factors, Oct4, Sox2, Klf4, and c-Myc, cooperatively reprogrammed reactive glial cells into iPSCs in the adult neocortex following TBI. These iPSCs further differentiated into a large number of neural stem cells, which further differentiated into neurons and glia *in situ*, and filled up the tissue cavity induced by TBI. The induced neurons showed a typical neuronal morphology with axon and dendrites, and exhibited action potential. Our results report an innovative technology to transform reactive glia into a large number of functional neurons in their natural environment of neocortex without embryo involvement and without the need to grow cells outside the body and then graft them back to the brain. Thus this technology offers hope for personalized regenerative cell therapies for repairing damaged brain.

In the United States (US) each year, 1.5 million civilian incidents of traumatic brain injury (TBI) occur and are graded from mild to severe^1^,^2^. Furthermore, it is estimated that more than 320,000 US veterans of the wars in Iraq and Afghanistan have sustained TBIs from blast waves of wartime improvised explosive devices (20% of 1.6 million)^3^,^4^. Traumatic brain injury is the leading cause of death in children and young adults^5^,^6^,^7^,^8^,^9^,^10^, and, for survivors, leaves many patients with substantial motor disability, cognitive impairment^7^,^8^,^11^, and epilepsy^12^,^13^,^14^,^15^,^16^. Due to the considerable damage and limited therapeutic approaches available, TBI is a serious public health problem.

Endogenous neural stem/progenitor cells (NSCs) were recently identified in the adult hippocampus and lateral ventricular zone^17^,^18^,^19^,^20^,^21^, but not in the adult neocortex of the mammalian brain, i.e. the neocortex is considered a non-neurogenic region^22^. This property presents a challenge to replace injured neurons in the neocortex where TBI imposes the most damage. However, hope is rekindled by the discovery that somatic mammalian cells can be epigenetically reprogrammed to induced pluripotent stem cells (iPSCs) through the exogenous expression of the Oct4, Klf4, Sox2, and c-Myc (OKSM)^23^,^24^,^25^. This novel technology has opened new therapeutic opportunities to generate stem cells in any area, including the neocortex, for cell replacement therapy in a number of disorders^23^,^24^,^25^,^26^.

TBI is a complex disease process caused by a cascade of systemic events. It not only causes cell death, but also activates glia by hypertrophy or proliferation^27^. Astrocytes and other glial cells such as microglia become activated in a process known as “reactive gliosis”^28^. Thus, a large number of proliferating glia are located around the injury area. Despite the controversial results on whether reactive glia mediates deterioration or protection of the nervous system after traumatic brain injury^29^,^30^,^31^, these glia represent a potential cellular resource for restoration of brain function following injury through reprogramming them into iPSCs *in vivo*. This strategy can be potentially developed for brain repair through reprogramming reactive glia resident in the injury area following TBI.

## Results

### Retroviral delivery of the four human factors Oct4, Klf4, Sox2, and C-Myc (hOKSM) reprogrammed reactive glia to embryonic stem-like cells *in vivo* following TBI

**Figure 1:**
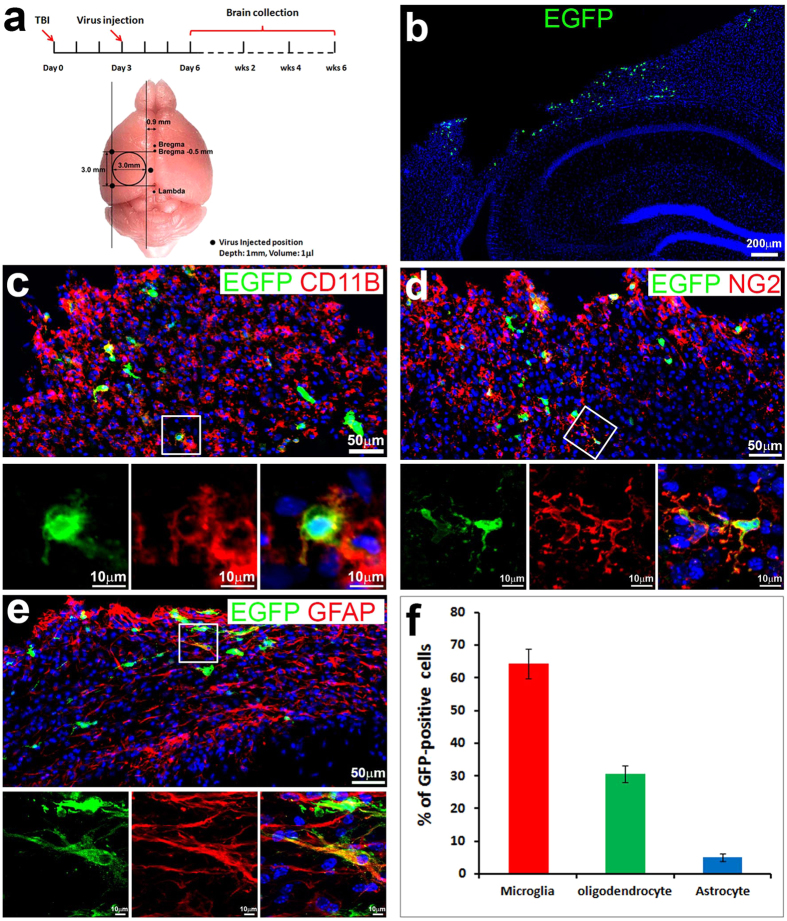
Retrovirus-infected reactive glia in cortex following moderate traumatic brain injury (TBI). (**a**) Strategy to induce pluripotent stem cells (iPSCs) *in vivo*. (**b**) 3 days after retrovirus injection, cells around the injured area were infected and started to express enhanced green fluorescent protein (EGFP). (**c**) EGFP-positive cells (green) colabeled with microglia specific marker, cd11b (red). (**d**) EGFP-positive cells (green) colabeled with oligodendrocyte progenitor cell marker, neural/glial antigen 2 (NG2, red). (**e**) EGFP-positive cells co-expressed astrocyte marker, glial fibrillary acidic protein (GFAP, red). (**f**) Quantification to show the ratio of EGFP-positive cells co-expressing different glia markers.

Induced pluripotent stem cells can be generated from somatic cells by ectopic expression of different transcription factors, originally Oct4, Klf4, Sox2, and c-Myc (OKSM)^32^. In order to program reactive glia to iPSCs *in vivo*, we used a retroviral system to express human OKSM (hOKSM) and enhanced green fluorescent protein (EGFP) as a cell-origin tracing marker in the injured brain. Retroviral dual cassette vectors either co-expressing the four hOKSM in combination with EGFP, or expressing only EGFP as control, were first tested for their infection and expression *in vitro* (Supplemental Figure 1). The mice at 12 weeks old received a head injury with a controlled cortical impact (CCI) at the moderate level (moderate TBI) as we previously reported^33^,^34^,^35^,^36^,^37^. The injury not only led to significant tissue lesion in the cortex (Supplemental Figure 2a), but also activated glial proliferation, which was pulse-labeled by 5-bromo-2′-deoxyuridine (BrdU) 4 h before sacrifice (Supplemental Figure 2). Most of the proliferating cells around the injury area are reactive microglia or neural/glial antigen 2 (NG2)-positive oliogodendrocyte progenitor cells at 3 days after moderate TBI (Supplemental Figure 2). We targeted hOKSM expression in the proliferating cells by direct intracranial injection of retroviruses expressing hOKSM-EGFP (1.6 × 10^7^pfu) 0.5~1.0 mm away from the epicenter in the ipsilateral cortex 3 days following moderate TBI (Fig. 1a). Three days after viral injection, coronal sections of brain containing the viral injected area were prepared and co-stained for EGFP, a marker for hOKSM expression, and different glia markers. There were 2464 ± 212 EGFP expressing cells dispersed around the epicenter in each mouse brain (Fig. 1b). We observed that 64.4 ± 4.6% of the infected cells were reactive microglia (Fig. 1c,f), and 30.6 ± 2.5% were NG2-positive oliogodendrocyte progenitor cells (Fig. 1d,f). Only a small percentage (5.1 ± 1.1%) of them were reactive astrocytes (Fig. 1e,f). The ratios of viral infection in each group were similar to their ratios of proliferation (Supplemental Figures 2e and 1f), suggesting that retroviruses infected proliferating cells without obvious cell type preference. These data indicated that the retrovirus mediated the delivery of hOKSM to the reactive glia, mainly reactive microglia, around the injury area following TBI.

**Figure 2:**
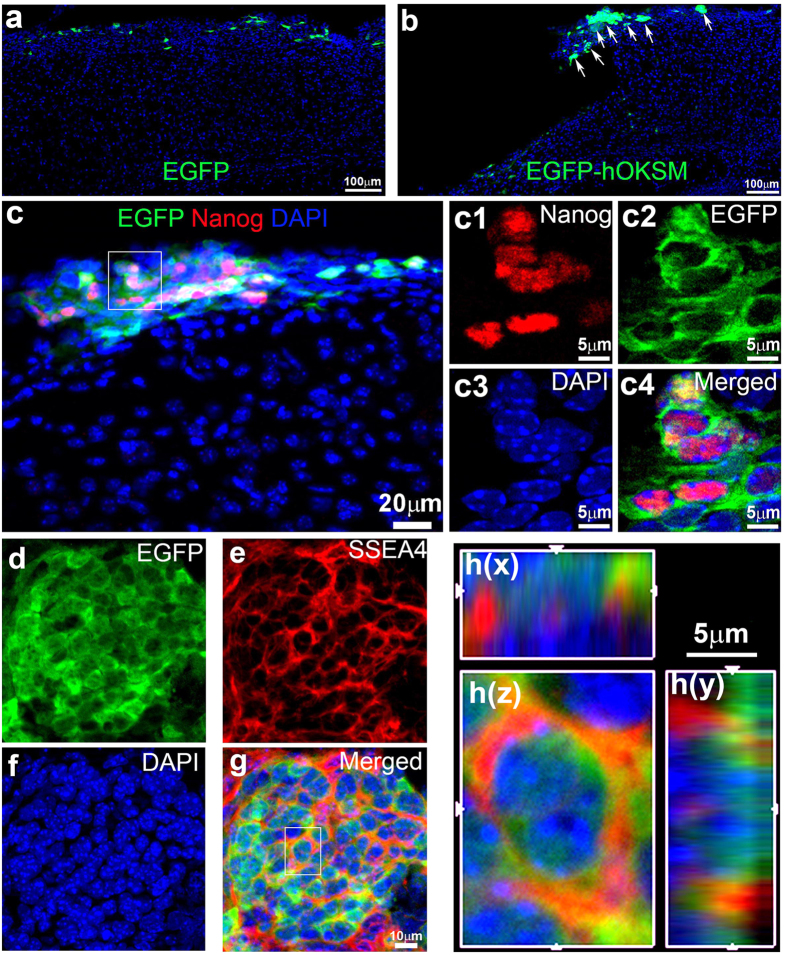
*In vivo* reprogramming reactive glia to embryonic stem-like cells. Tracing the fates of retrovirus infected cells in the injured cortex 2 weeks and 4 weeks after traumatic brain injury (TBI) with immunostaining. (**a**) Cells expressing EGFP (green) around the injury area 2 weeks after TBI. (**b**) Cell clusters (white arrows) expressing 4 human transcriptional factors (hOCT4, hKLF4, hSOX2 and hcMYC, hOKSM) and EGFP (green) around the injury area 2 weeks after TBI. (**c**) Cells expressing hOKSM-EGFP (green) also colabeled with Nanog (red). c1–4. Images of single focal section images to show colabeling of hOKSM/EGFP (green) with Nanog (red) in the cells within the white box from panel (**c)**. (**d–g)** hOKSM/EGFP (green) expressing cells form cluster and expressed SSEA4 4 weeks after TBI. (**h**(x–z)). Images of three-dimensional reconstruction to confirm that a hOKSM/EGFP expressing cell in the white box of panel (**g**) also expressed SSEA4.

To determine whether over-expression of hOKSM in the reactive glia could reprogram them into iPSCs *in vivo* following TBI, the TBI-injured mice received either reprogrammed retrovirus expressing hOKSM-EGFP, or control retrovirus expressing only EGFP. Two weeks after TBI, the mice were sacrificed to assess the viral infected cells with immunostaining (Fig. 2). In the control mice in which control retrovirus expressing only EGFP was injected, there were 1736 ± 184 EGFP-positive cells. The number of EGFP-positive cells was reduced, while the distribution pattern remained similar as single cells and dispersed around the epicenter (Fig. 2a). Nonetheless, in the mice receiving injection of retrovirus expressing hOKSM-EGFP, the EGFP-positive cells expressing hOKSM showed cell clusters (Fig. 2b, white arrows), which were not seen in the control mice, suggesting they were expanding.

Next we assessed whether these expanding cells in the cell colony expressed embryonic stem cell marker with immunostaining. To avoid false positives from overlaying cells due to the thickness of the brain sections, we detected the Nanog expression in the EGFP-positive cells at the single focal section with a Zeiss microscope equipped with an apotome. The EGFP-positive cells in the cluster strongly expressed Nanog (Fig. 2c, and c1–c4), a marker for embryonic stem cells, which was not seen in the control mice expressing only EGFP. At 4 weeks after TBI, the size of EGFP-expressing cell clusters became bigger (Fig. 2d) compared to cell clusters seen at 2 weeks after TBI (Fig. 2b), indicating the active expansion of cells expressing hOKSM-EGFP. The proliferating cells had filled up the cavity caused by the TBI-induced tissue lesion. There was no cavity observable in the ipsilateral brain hemisphere (Supplemental Figure 3). The hOKSM-EGFP-expressing cells in the cluster also expressed the stage-specific embryonic antigen-4 (SSEA4), a cell surface marker expressed in the embryonic stem cells (Fig. 2d–g). Images of single cells with 3-dimensional reconstruction also demonstrated the co-localization of SSEA4 with the hOKSM-EGFP-expressing cells (Fig. 2h(z), h(x), and h(y)). In contrast, the expression of SSEA4 was undetectable at 2 weeks after TBI (Supplemental Figure 4). These results indicate the hOKSM-EGFP expressing cells were reprogrammed to embryonic stem-like cells in the adult brain following TBI. A total of 36 ± 2 EGFP cell clusters was observed in each mouse, which indicated that about 1.5% of the hOKSM-EGFP cells were forming cell clusters 2 weeks after TBI. This efficiency of colony formation *in vivo* is extremely high compared to ~0.01–0.1% of the regular reprogramming rate *in vitro*^38^. These data suggest that retrovirus-mediated ectopic expression of hOKSM can efficiently reprogram reactive glia into embryonic stem-like cells in their natural environment following TBI.

### Reprogrammed embryonic stem-like cells produced cells in all three embryonic germ layers and then mainly differentiated towards neural fate in the injured brain

**Figure 3:**
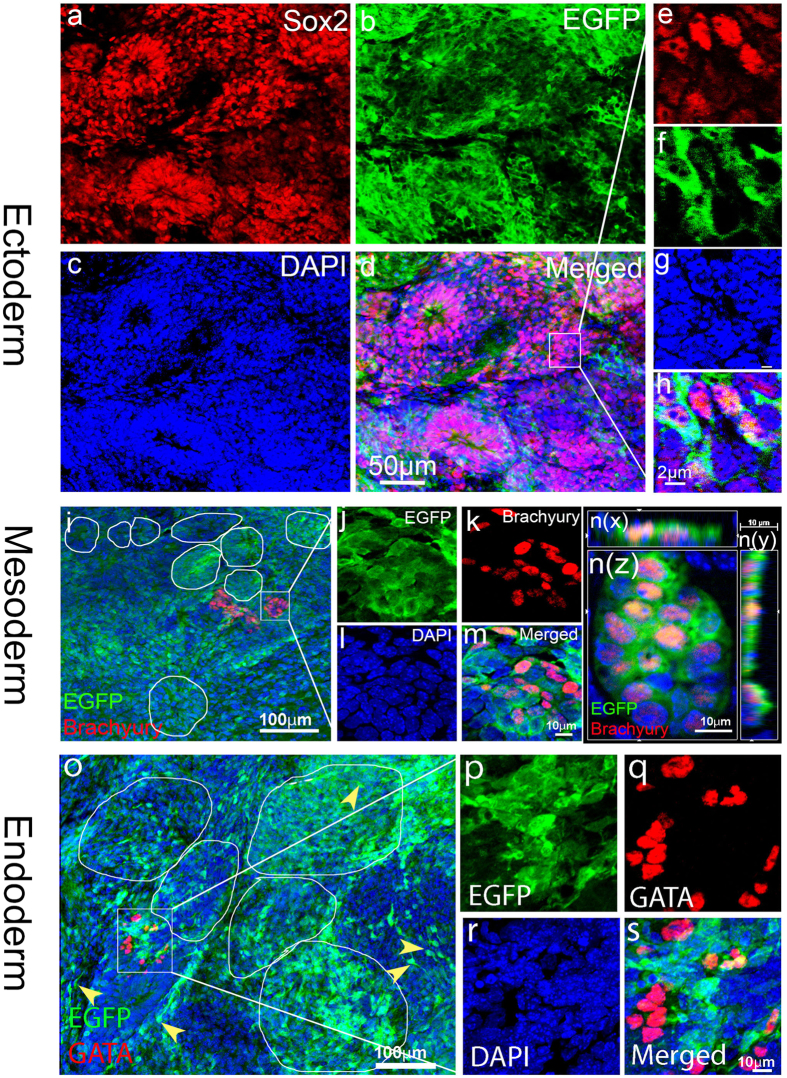
Reprogrammed glia produced cells in all three embryonic germ layers at 4 weeks after TBI. Cells expressing hOKSM-EGFP expanded into cellular clusters and expressed markers of all three embryonic germ layers in the injured cortex at 4 weeks after traumatic brain injury (TBI). (**a–d**) A large number of hOKSM-EGFP expressing cells (green) in the clusters highly expressed Sox2 (red), a marker for embryonic ectoderm. (**e–h**) Images of single focal section to show colabeling of hOKSM-EGFP (green) with Sox2 (red) in the cells within the white box from panel (**d**). (**i**) A small number of the hOKSM-EGFP expressing cells expanded into cell clusters and expressed brachyury, a marker of embryonic mesoderm. White circles indicate neural tube-like structures formed by EGFP-positive cells. (**j–m**) Enlarged images of cells within the white box in the panel (**i**). (**n**(**x–z**)) Images of three-dimensional reconstruction to confirm that some of the hOKSM-EGFP expressing cells in the white box of panel (**i**) also expressed Brachyary. (**o**) A small number of the hOKSM-EGFP expressing cells expanded into cell clusters and expressed Gata4, a marker of embryonic endoderm. (**p–s**) Enlarged images of cells within the white box in the panel (**o**). White circles indicate neural tube-like structures formed by EGFP-positive cells. Yellow arrowheads pointed out the reprogrammed cells with long processes.

To determine whether these reprogrammed embryonic stem-like cells have pluripotency and can differentiate into cells in all three embryonic germ layers, we immunostained the tissue with antibodies against markers of ectoderm (SRY (sex determining region Y)-box 2, (Sox2)), mesoderm (Brachyury), or endoderm (Gata4)^39^. At 4 weeks after TBI, we again observed a large number of EGFP-positive cells in the injury brain injected with retroviruses expressing hOKSM-EGFP (Fig. 3b). A majority (>85%) of the EGFP-positive cells formed tube-like structures and highly expressed Sox2 (Fig. 3a–h). Sox2 is a transcriptional factor that is essential for maintaining self-renewal or pluripotency of undifferentiated embryonic stem cells^40^,^41^. It is worth noting that the expression of Sox2 in the EGFP-cell clusters was undetectable using immunostaining at 2 weeks after TBI (Supplemental Figure 5). These data indicated that the expression of Sox2 in the EGFP-positive cells at 4 weeks after induction was not retrovirus-mediated ectopic expression; it was from the reprogrammed cells.

Occasionally we observed a small number of Brachyury-positive cells (<0.1% of total EGFP-positive cells) residing between the tube-like structures, similar to neural tubes during early embryonic development (Fig. 3i). Images of a single focal section with a confocal image (Fig. 3j–m) and three-dimensional (3-D) reconstruction (Fig. 3n(z), n(x), and n(y)) showed that Brachyury was expressed in the hOKSM-EGFP-positive cells. We rarely observed Gata4-positive cells (<0.01% of total EGFP-positive cells) in the injured brain that received retrovirus injection (Fig. 3o). Images of a single focal section using a microscope equipped with aptome showed that Gata4 was expressed in the EGFP-positive cells (Fig. 3p–s). Furthermore, some of the EGFP-positive cells began to exhibit neuronal morphology with long processes (Fig. 3o, shown by arrowheads in yellow). These data indicate that these reprogrammed embryonic stem-like cells are multipotent and can differentiate into cells in all three embryonic germ layers, suggesting that ectopic expression of hOKSM reprogrammed reactive glia into iPSCs in the neocortex following TBI. These iPSCs mainly differentiated into cells in the ectoderm in the injured brain.

**Figure 4:**
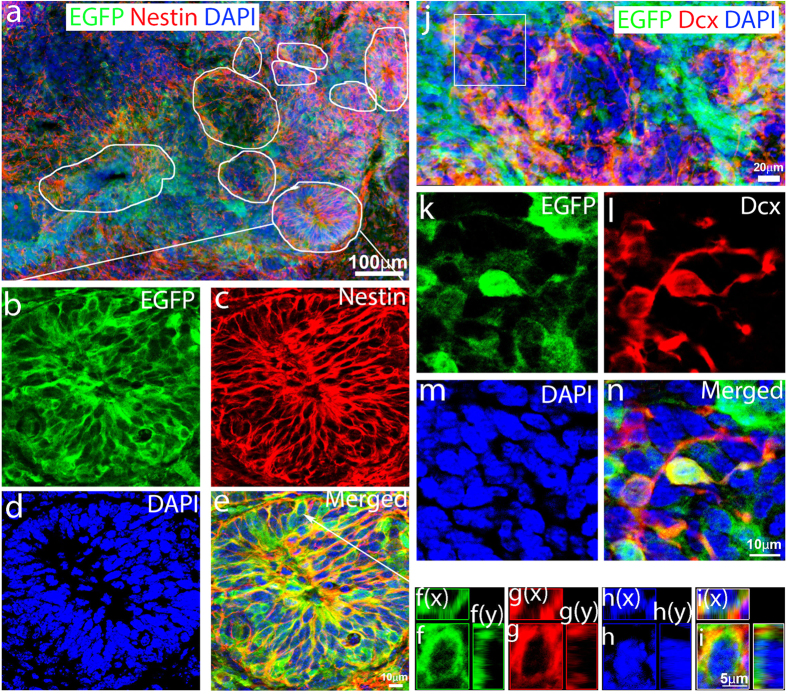
Reprogrammed cells differentiated into neural fate at 4 weeks after traumatic brain injury (TBI). (**a**) hOKSM-EGFP expressing cells formed neural tube-like structures (white circle) in the injured cortex. (**b–e**) EGFP-positive cell cluster (EGFP) exhibited typical neural tube-like structure and expressed Nestin (red), a marker used for neural stem cells. (**f–h**) Images of three-dimensional reconstruction to confirm that a hOKSM-EGFP expressing cell in the panel e also expressed Nestin. (**j**) Some of the hOKSM-EGFP expressing cells (green) started to differentiate into doublecortin (Dcx)-positive immature neurons (red). (**k–n**) Enlarged images of cells in the white box of panel (**j**) to show the hOKSM-EGFP expressing cells also expressed Dcx, a marker for immature neurons.

Since most of the EGFP-positive cells with ectopic expression of hOKSM-EGFP formed large numbers of neural tube-like structures at 4 weeks after TBI, we determined whether they were neural stem cells, and whether they could produce new neurons *in vivo* in the injured neocortex, a non-neurogenic region in the adult brain. EGFP was used as a lineage-tracing marker to detect the fates of the reprogrammed iPSCs. We found that hOKSM-EGFP-positive cells expanded dramatically from 2 weeks to 4 weeks following viral injection (Figs 2 and 3) and (Fig. 4a). They formed neural tube-like structures and filled up the cavity in the brain caused by the tissue lesion at 4 weeks after TBI (Fig. 4a). We further confirmed the neural differentiation of iPSCs using double immunostaining with antibodies against EGFP and Nestin, a widely used marker for neural stem cells^42^,^43^ (Fig. 4a–e). We confirmed that a large number of EGFP-positive cells expressed Nestin and clustered as neural tube-like structures (Fig. 4a–e). Three-dimensional reconstruction (Fig. 4f(z), f(x), and f(y)) showed that Nestin was expressed in the EGFP-positive cells. These data strongly support the notion that glia-reprogrammed iPSCs can mainly differentiate into neural stem cells in the injury environment following traumatic brain injury. To further determine whether these neural stem cells can differentiate into new neurons in the neocortex, a non-neurogenic region, *in vivo*, we performed double immunostaining with antibodies against EGFP and progressive markers of neuronal differentiation. The results showed that some of the EGFP-positive cells displayed immature neuron morphology with long processes and expressed doublecortin (Dcx) (Fig. 4i–n), a widely used marker for immature neurons^44^,^45^, indicating that new neurons are produced in the neocortex.

**Figure 5:**
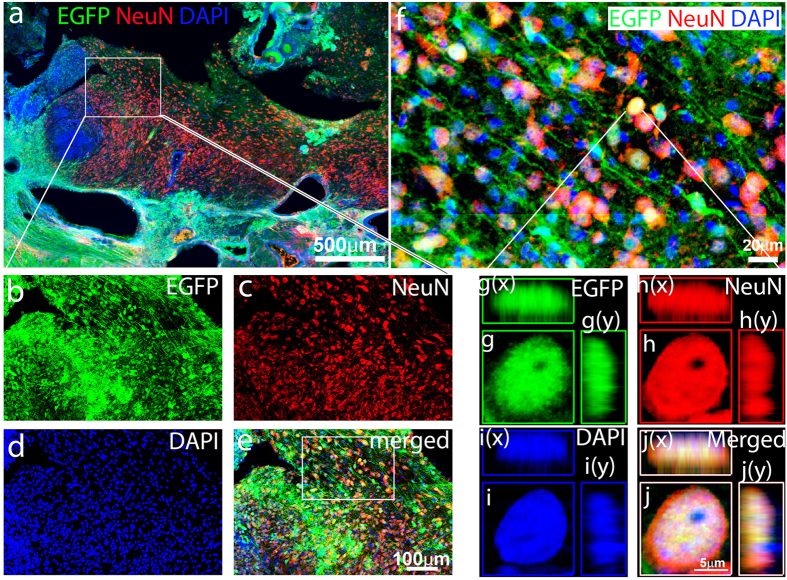
Reprogrammed cell-derived immature neurons developed into mature neurons at 6 weeks after traumatic brain injury (TBI). (**a**) The hOKSM-EGFP expressing cells (green) differentiated into a large number of mature neurons expressing neuronal nuclei protein (NeuN, red), a marker for mature neurons, in the injured cortex. (**b–e**) Enlarged images from pane a to show hOKSM-EGFP expressing cells (green) colabeled with NeuN (red). (**f**) Enlarged image from pane (**e**) to show individual neurons with long processes expressing both EGFP (green) and NeuN (red). (**g–j**) Images of three-dimensional reconstruction to confirm that a hOKSM-EGFP expressing cell in the panel (**f**) also expressed NeuN.

We further found that a large number of EGFP-positive cells expressed the mature neuron markers, NeuN^46^ (Fig. 5) and Map2^47^ (Supplemental Figure 6), at 6 weeks after TBI. The reprogramming-generated mature neurons displayed parallel processes along the same orientation (Fig. 5f). To avoid false positives from overlaying cells due to the thickness of the brain sections, we detected the NeuN expression in the EGFP-positive cells at the single focal image and 3-D reconstruction with images from a microscope equipped with an apotome (Fig. 5g–j). We further confirmed the co-labeling of NeuN with EGFP-positive cells. In contrast we could not detect NeuN expression in the reprogrammed cells at 4 weeks after TBI (Supplemental Figure 7). Collectively, these studies suggest that *in vivo* programmed iPSCs can differentiate into a large number of mature neurons, we named them iNeurons, in the neocortex following TBI.

### *In vivo* iNeurons electrically matured and integrated into the neural network

**Figure 6:**
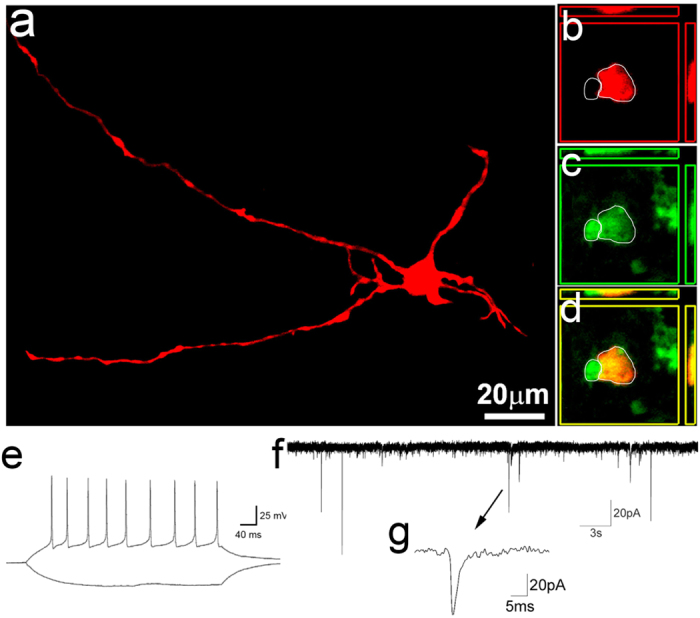
Functional integration of iNeurons. (**a–d**) Confocal images of recorded EGFP-positive cells, which were also loaded with biocytin during recording. (**e–g**) Electrophysiology of a EGFP-positive cell labeled in panel (**a**). This cell fired repetitive action potentials in response to depolarizing currents (**e**) and showed spontaneous synaptic currents when voltage clamped at the resting membrane potential (**f,g**).

To determine whether the mature iNeurons were functional, we sacrificed the mice and sliced the brains for electrophysiological recording 6 weeks after TBI. Electrophysiology was performed on EGFP-positive cells in the adult live brain slices to examine the functionality of iPSC-derived mature iNeurons using whole-cell patch-clamp recordings. It is known that immature neurons rarely fired repetitive action potentials when stimulated. Most patched cells exhibited trains of action potentials and large inward currents following depolarization, indicating functional voltage-gated sodium channels (Fig. 6). In addition, most reprogrammed cells that were recorded showed spontaneous synaptic currents indicating the presence of postsynaptic receptors that formed functional synapses. After the recordings, cells were loaded with biocytin to visualize the morphology. The biotin filling revealed typical neuronal morphologies of recorded cells (Fig. 6a). EGFP immunostaining was used to confirm that the cells were derived from iPSCs (Fig. 6b–d). We further found that there is a EGFP-positive cell located closely to the recorded mature neurons on the left (Fig. 6c,d). This cell is small in body size, and is likely a glial cell derived from the iPSCs. Together, these data suggest that the glia-reprogram-derived mature neurons showed electrophysiological activities and typical neuronal morphology *in vivo*, indicating iPSC-derived iNeurons are functional in an injured environment.

**Figure 7:**
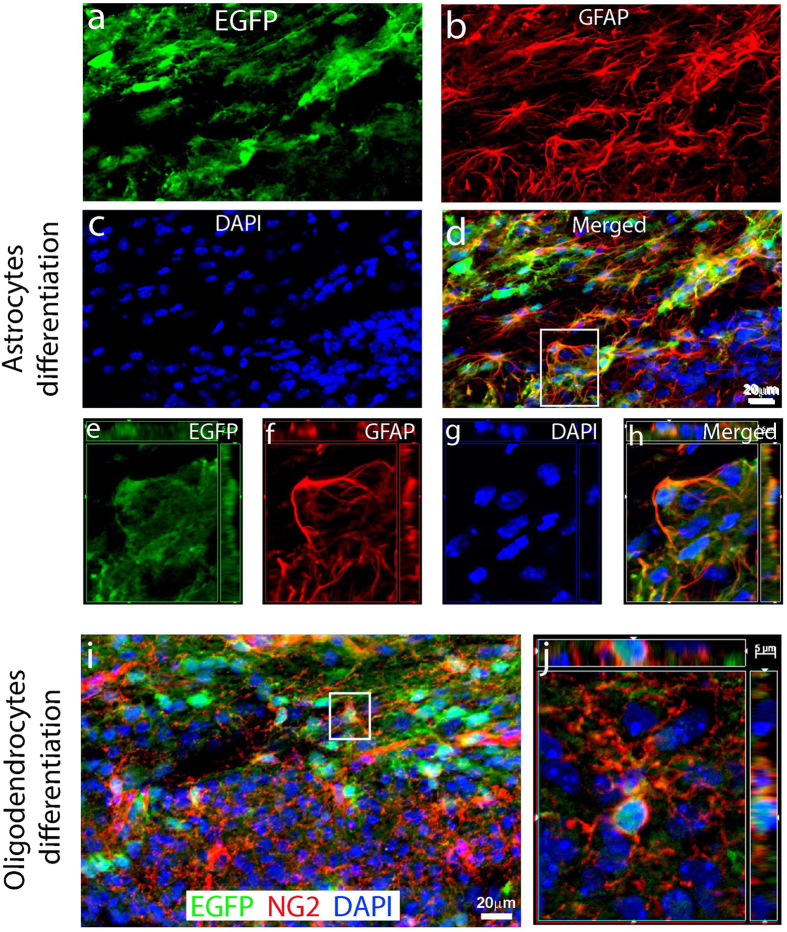
Reprogrammed cells also differentiate into glia at 6 weeks after traumatic brain injury (TBI). (**a–d**) Some of the hOKSM-EGFP expressing cells (green) differentiated into astrocytes expressing GFAP (red) in the injured cortex at 6 weeks after TBI. (**e–h**) Images of three-dimensional reconstruction to confirm that a hOKSM-EGFP expressing cell in the panel (**d**) also expressed GFAP. (**i**) A subpopulation of the hOKSM-EGFP expressing cells (green) differentiated into oligodendrocyte precursors expressing NG2 (red) in the injured cortex. (**j**) Images of three-dimensional reconstruction to confirm that a hOKSM-EGFP expressing cell in the panel (**i**) also expressed NG2.

The brain contains billions of cells, neurons, and glial cells that assemble into a highly refined structure. Brain function might actually rely on the coherent activity of neuron–glia networks. To assess whether reprogrammed iPSCs can also differentiate into glial cells and repopulate the injured cortex following TBI, we examined the glial differentiation of reprogrammed iPSCs based on EGFP as a lineage-tracing marker. In the central nervous system, there are three types of glial cells: astrocyte, microglia, and oligodendrocyte. Therefore, we performed double immunostaining with antibodies against EGFP and each glial cell marker. The results showed that a large number of EGFP-positive cells expressed GFAP, a marker for astrocytes^48^, at 6 weeks after TBI (Fig. 7a–d). Confocal images with 3-D reconstruction further confirmed the result (Fig. 7e–h). Double immunostaining with antibodies against EGFP and NG2, a marker for oliogodendrocyte progenitor cells^49^, also showed that a large number of EGFP-positive cells expressed NG2 (Fig. 7i,j).

We next assessed whether iPSCs differentiate into microglia. We found a large number of CD-11b-positive microglia in the peri-lesion region, while we saw only a very small number of CD-11b-positive cells in the tissue lesion region, which was filled with a large number of EGFP-positive cells at 4 weeks after TBI (Supplemental Figure 8a–d). There were no EGFP-positive cells co-labeled with CD-11b. At 6 weeks after TBI, we found more CD-11b-positive cells in the tissue lesion region (Supplemental Figure 8g,h), but none of them were co-labeled with EGFP (Supplemental Figure 8i–l). These results indicate that these CD-11b-positive cells were not derived from reprogrammed iPSCs; instead, they were endogenous microglia that migrated into the injury area. Together these data indicate that reprogrammed iPSCs can also produce a large number of glial cells, preferentially astrocytes and oligodendrocytes, but not microglia, in the injured cortex. Furthermore, the mice developed teratoma in the brain if they were allowed to survive more than 8 weeks after TBI (Supplemental Figure 9).

## Discussion

Our results demonstrate that four transcription factors, Oct3/4, Sox2, Klf4, and c-Myc, can reprogram glial cells into iPSCs in the brain following TBI. The reprogrammed iPSCs preferentially produce large numbers of functional neurons and glial cells. The lesion cavity was completely diminished after reprogramming.

Traumatic brain injury induced with a controlled cortical impact usually causes significant cell death and even results in tissue lesion in the mouse cortex. There is no FDA-approved medicine available to stop cell death in the brain following TBI; furthermore, the neocortex is a non-neurogenic region^22^. However, an extremely large number of proliferating glia, including astrocytes and microglia, locate around the injury area. After the acute phase, the reactive astrocytes will form scar tissue in the brain. Here we reprogram a small number of glia into functional neurons in an originally non-neurogenic region for possible brain repair following TBI. This *in vivo* reprogramming technology with forced expression of four transcription factors, Oct3/4, Sox2, Klf4, and c-Myc, transforms reactive glia into functional neurons. Sox2 alone has been shown to be sufficient to reprogram resident astrocytes into proliferative neuroblasts in the adult mouse brain^50^. Ectopic expression of NeuroD1 was able to reprogram astrocytes into glutamatergic neurons, while reprogramming NG2 cells into glutamatergic and GABAergic neurons^51^. These studies provide novel approaches to convert non-neurogenic cortex into a neurogenic region.

Neuronal loss is a prominent pathological feature of many neurological disorders such as Alzheimer’s disease, Huntington’s Diseases, stroke, and TBI. These disorders induce different levels of cell death in different regions of the brain. For example, both TBI and stroke cause large number of neurons death. A very large number of new neurons are required to replace the damaged neurons. In comparison to two other approaches, our approach with forced expression of four transcription factors has the advantage of generating a large number of mature neurons in the TBI-injured brain (Fig. 5). However, the disadvantage of this approach is that the induced stem cells or precursor cells overgrow and may develop into a tumor.

There is no exogenous cell transplantation involved in this *in vivo* reprogramming approach. Neural transplantation has been recognized to be a valuable technique for replacement of lost cellular populations and reconstruction of local neuronal circuitry. However, scientists have been facing big challenges that include possible pathogen contamination when recreating the cells in petri dishes, delivery, maturation, rejection, and integration. Our method of reprogramming *in vivo* restores neurons without removing targeted cells from their natural environment and without the need for a culture in a petri dish. The approach proposed in this study is all *in vivo*. No exogenous cell transplantation is required. This approach avoids the complications that are caused by exogenous cells, such as contamination and immune rejection. Furthermore, there are large numbers of new induced neurons with long processes lining up in the injured region of the cortex, which were not seen in the brain following neural stem cell transplantation. This result suggests that the induced new neurons generated *in situ* from reprogramming have a much better survival rate and a higher capability to integrate into the existing neural network.

Similar reprogramming approaches have been used to generate other cell types *in vivo*, such as pancreatic beta-cells with 3 transcriptinoal factors, Ngn3, Pdx1, and Mafa, for treating diabetes^52^, and cardiomyocytes for heart repair following heart failure^53^. Forced expression of four other transcriptional factors, Gata4, Hand2, Mef2C, and Tbx5, in cardiac fibroblasts in mice reprograms these cells into functional cardiac-like myocytes^53^,^54^,^55^. *In vivo* cell reprogramming technology provides a novel approach to derive different types of new cells directly from a patient’s somatic cells without embryo involvement. Thus, this novel approach overcomes ethical concerns and is minimally invasive.

TBI causes significant cell death^56^,^57^,^58^,^59^ and tissue lesion in the neocortex^60^,^61^, leaving many patients with substantial motor disability and cognitive impairment^11^,^62^. Although the *in vivo* reprogramming approach in this study has the advantage of generating a large number of mature neurons in the TBI-injured brain, tumorigenesis prevents its possible application in the translational research. Any strategies to avoid this potential for cell overgrowth will accelerate efforts to develop a novel regenerative therapy by both increasing neural integration and avoiding the need to grow cells outside the body and then graft them back into the brain.

The retroviral system is used to produce the first iPSCs and it is still one of the most effective approaches by far to produce iPSCs from somatic cells. A retroviral vector can integrate into the host chromosome and constitutively express the carried genes in the host cells. The constitutive expression of OSKM, which are four very important transcriptional factors, can efficiently reprogram the hosted cells into iPSCs. Meanwhile, continuous expression of these transcriptional factors in the iPSCs may also lead to their proliferation out of control and turn themselves into tumor cells. An approach to avoid teratoma formation is major technical and scientific obstacles that remain to be overcome before cell therapy based on iPS cells becomes a realistic therapeutic modality. Numerous approaches have been developed in an attempt to block teratoma/tumor formation, including the introduction of suicide genes^63^, selecting the desired cell type^64^, immunodepletion^65^, or introducing cytotoxic antibody^66^. Recently, Lee *et al.* reported a small molecule that can inhibit pluripotent stem cell-derived teratoma formation^67^. However, a clinically viable strategy to eliminate teratoma formation remains to be developed^68^.

In summary, our results report a strategy to reprogram glia into neurons and convert a non-neurogenic cortex into a neurogenic region. Reprogramming reactive glia into iPSCs *in vivo* to produce new neurons in their natural environment of the cortex may suggest a strategy for brain repair following TBI and other neurodegenerative diseases.

## Materials and Methods

### Animal care

Male C57 BL/6 mice (Jackson Laboratories) were group-housed and kept in a 12/12-hour light/dark cycle with free access to food and water ad libitum. The animals were used in experiments at an age of 12 weeks. All procedures were performed under protocols approved by the Indiana University Animal Care and Use Committee. All experiments were performed in accordance with guidelines and regulations of Indiana University Biosafety Committee.

### Controlled cortical impact traumatic brain injury

12-week-old mice were subjected to moderate controlled cortical impact injury (CCI) as previously described^33^,^34^,^36^,^59^,^69^,^70^. Briefly, the mice were anesthetized and placed in a stereotaxic frame (Kopf Instruments, Tujunga, CA) prior to TBI. Using sterile procedures, the skin was retracted and a 4 mm craniotomy centered between the lambda and bregma sutures was performed. A point was identified midway between the lambda and bregma sutures and laterally midway between the central suture and the temporalis muscle. The skullcap was carefully removed without disruption of the underlying dura. Prior to injury induction, the tip of the impactor was angled and kept perpendicular to the exposed cortical surface. The mouse CCI model uses an electromagnetic impactor that allows one to alter the severity of the injury by controlling contact velocity and the level of cortical deformation independently. In the experiments for this study, the contact velocity was set at 3.0 m/s and deformation at 1.0 mm. These settings will result in an injury of moderate severity. During surgery and recovery, the core body temperature of the animals was maintained at 36–37 °C using a heating pad.

### Retrovirus preparation and injection

Concentrated retroviral solution (1.6 × 10^7^ pfu/ml), which expressed human Oct4, KLF4, SOX2, cMYC, and green fluorescent protein (GFP), was prepared as reported previously^20^. Retrovirus was stereotactically injected into the cortex 3 days after TBI. A Hamilton injector was used to deliver retrovirus (1 μl) at 3 sites and at a depth of 1.0 mm (Fig. 1a).

### Tissue processing

The animals were deeply anesthetized and then perfused transcardially with saline, followed by a fixative containing 4% paraformaldehyde (PFA) in PBS. The brains were removed, postfixed overnight in PFA, and cryoprotected for 48 h in 30% sucrose. Serial 30 μm thick coronal sections were cut using a cryostat (LeicaCM 1950) and stored at −20 °C. The sections were then processed for immunohistochemical analysis.

### Immunohistochemistry

Free floating sections were washed 3 times in PBS, and then incubated in blocking solution (0.1% Triton X-100, 1% bovine serum albumin, and 5% normal serum in PBS) for 1 h at room temperature, followed by overnight incubation with primary antibody at 4 °C. The sections were washed again with PBS (3 times), and incubated at room temperature for 2 h with the secondary antibody. After treatment (2 min) with DAPI (4′,6-diamidino-2-phenylindole), the sections were washed with PBS (3 times). The sections were put on the slides and mounted using Fluorescent Mount G. Primary antibodies and their final concentrations were as follows: anti-EGFP (1:1000, chicken, Abcam), anti-Nanog (1:200, rabbit, Abcam), anti-SSEA4 (1:100, mouse, Abcam), anti-nestin (1:1000; rabbit; Covance), anti-GFAP (1:1000, mouse, Sigma), anti-CD11b (1:200, rabbit, Millipore), anti-IbaI (1:200, goat, Abcam), anti-NG2 (1:200, rabbit, Millipore), anti-Dcx (1:1000, Guniea pig, Millipore), anti-NeuN (1:1000, mouse, Millipore), anti-Brachyury (1:100, rabbit, Abcam), anti-Gata4 (1:100, rabbit, Abcam). Secondary antibodies from Jackson ImmunoResearch Laboratories, Inc. were applied in a dilution of 1:1000.

### Hematoxylin and Eosin staining

The Hematoxylin and Eosin staining (H&E) was performed to the brain histology. Briefly, the slides with tissue sections mounted were rinsed in distilled water for 5 minutes, and then stained in hematoxylin (Harris Hematoxylin, Sigma, HHS-32) for 6 minutes. After rinsed in running tap water for 20 minutes, the sections were decolorized in acid alcohol for several seconds. The slides were rinsed well in tap water for 5 minutes before immersed in Lithium Carbonate for 3 seconds, and then rinsed again for 5 minutes. After counterstained in Eosin (1% aqueous Eosin-Y) for 15 seconds, the sections were dehydrated by EtOH 95% (3 minutes) and EtOH 100% (3 minutes). Followed by clear in Xylene I and II (5 minutes), the slides were mounted with DPX in fume hood.

### Cell counting

Immunohistochemistry was performed simultaneously on sections to detect the target cells. For total infected cell number, series of every sixth section (30 μm thickness, 180 μm apart) through injured cortex were processed with antibody against EGFP. The cell total number was determined through a blinded quantitative histological analysis. The profile count method was used. Every single EGFP-positive cell throughout the entire 30 μm section was counted under a fluorescent microscope using the 40× objective through a whole series of sections, and then 6 times to get the total number. For cell type specification, 3 epicenter sections were selected to do the immunostaining. The double-labeled cell was determined as follows. We used EGFP as an indicator. When the EGFP-positive cell showed in the frame, we switched to the channel matching the cell specific marker. If the target cell also had been marked, we considered it as double-labeled cell. The double-labeled cells were shown as a percentage compared to the total EGFP-positive cells.

### Microscopy

The sections were analyzed using an inverted microscopy system (Zeiss Axiovert 200 M) combined with apotome and interfaced with a digital camera (Zeiss Axio Cam MRc5) controlled by a computer. Images were captured using apotome in software (AxioVision, v4.8) and then assembled and labeled in Photoshop 7.0 (Adobe Systems).

### Electrophysiological recording

Mice were deeply anaesthetized and transcardially perfused with chilled (4 °C) artificial cerebrospinal fluid (ACSF: 119 mM NaCl, 2.5 mM KCl, 2 mM MgSO4, 2 mM CaCl_2_, 26 mM NaHCO_3_, 1.25 mM NaH_2_PO_4_, 10 mM D-glucose at pH 7.4, 300 mOsm, and aerated with 95% O_2_/5% CO_2_) including 0.5 mM CaCl_2_, 5 mM MgCl_2_ and 1% kynurenic acid. Acute slices (350 μm thickness) were prepared from adult mice (4 months old) at 6 wpi with a Leica VT1200S slicer. Before recording, brain slices were incubated in aerated artificial cerebrospinal fluid (ACSF) at 35 °C, followed by at least 1 h at room temperature. A single slice was then transferred to a submersion chamber and perfused at 3 mL  min^−1^ with aerated ACSF at room temperature. Lineage-traced cells in each slice were identified under visual guidance using IR-DIC optics and GFP fluorescence. Whole-cell current-clamp and voltage-clamp recordings were performed using glass pipettes (~5–9 MΩ) filled with intracellular solution (0.2 mM EGTA, 130 mM K-gluconate, 6 mM KCl, 3 mM NaCl, 10 mM HEPES, 4 mM ATP-Mg, 0.4 mM GTP-Na and 14 mM phosphocreatine-Tris at pH 7.2 and 295 mOsm). For biocytin labeling, intracellular solution was supplemented with 2% biocytin (Sigma, B4261). All recordings were obtained with a MultiClamp 700B amplifier. Currents were filtered, acquired, and digitized at 10 kHz using Clampex10.3 (Molecular Devices). Action potentials were recorded in current clamp mode and elicited by a series of current injections starting from −200 pA with 50 pA step increments and 500 ms in duration. Series and input resistance were measured in current clamp mode with a 500 ms, −10 mV step from a −70 mV holding potential (filtered by 10 kHz, sampled at 50 kHz). Cells were accepted only if the series resistance was less than 15 MΩ and stable throughout the experiment. Spontaneous synaptic currents were recorded in voltage clamp mode. In all voltage clamp recordings, cells were clamped at −60 mV or −80 mV, whichever is close to the resting membrane potential of the cell, except during the voltage step protocol. In all current clamp recordings, recordings were made at the resting membrane potential or without any current injection. Data analysis was performed in Clampfit10.3 (Molecular Devices).

## Additional Information

**How to cite this article**: Gao, X. *et al.* *In vivo* reprogramming reactive glia into iPSCs to produce new neurons in the cortex following traumatic brain injury. *Sci. Rep.* **6**, 22490; doi: 10.1038/srep22490 (2016).

## Supplementary Material

Supplementary Information
