# Supplementary material for: In vivo reprogramming reactive glia into iPSCs to produce new neurons in the cortex following traumatic brain injury
Source: Sci Rep. 2016 Mar 9;6:22490. doi: 10.1038/srep22490 (PMC4783661; doi:10.1038/srep22490)
Supplement: Supplementary Information [file srep22490-s1.pdf]

In vivo reprogramming reactive glia into iPSCs to produce new neurons in the cortex following traumatic brain injury

Xiang Gao[1], Xiaoting Wang[1], Wenhui Xiong[1], Jinhui Chen[1, *]

[1] Spinal Cord and Brain Injury Research Group, Stark Neuroscience Research Institute, Department of Neurosurgery, Indiana University. 320 W 15th Street, Indianapolis, IN 46202

Supplemental figure 1

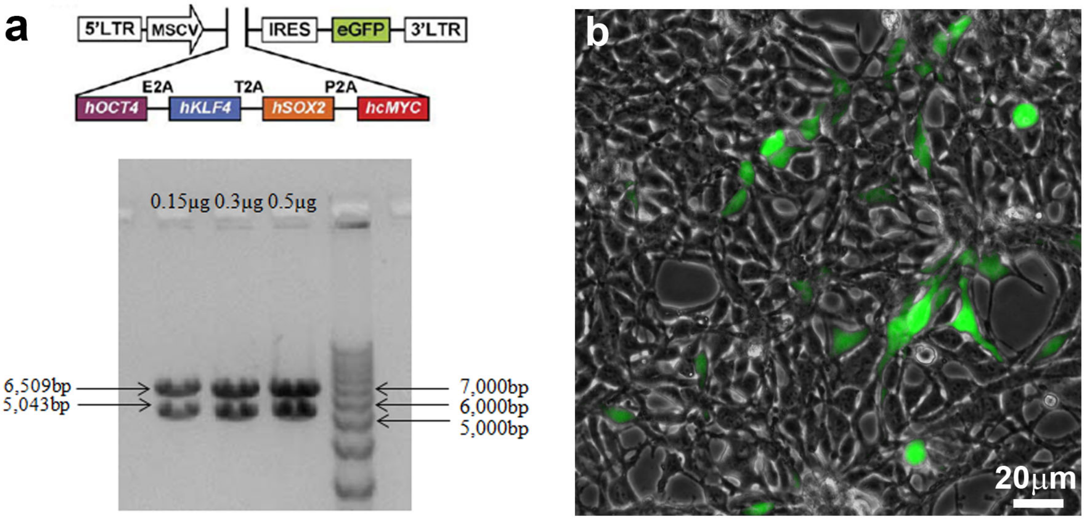

Supplemental figure 2

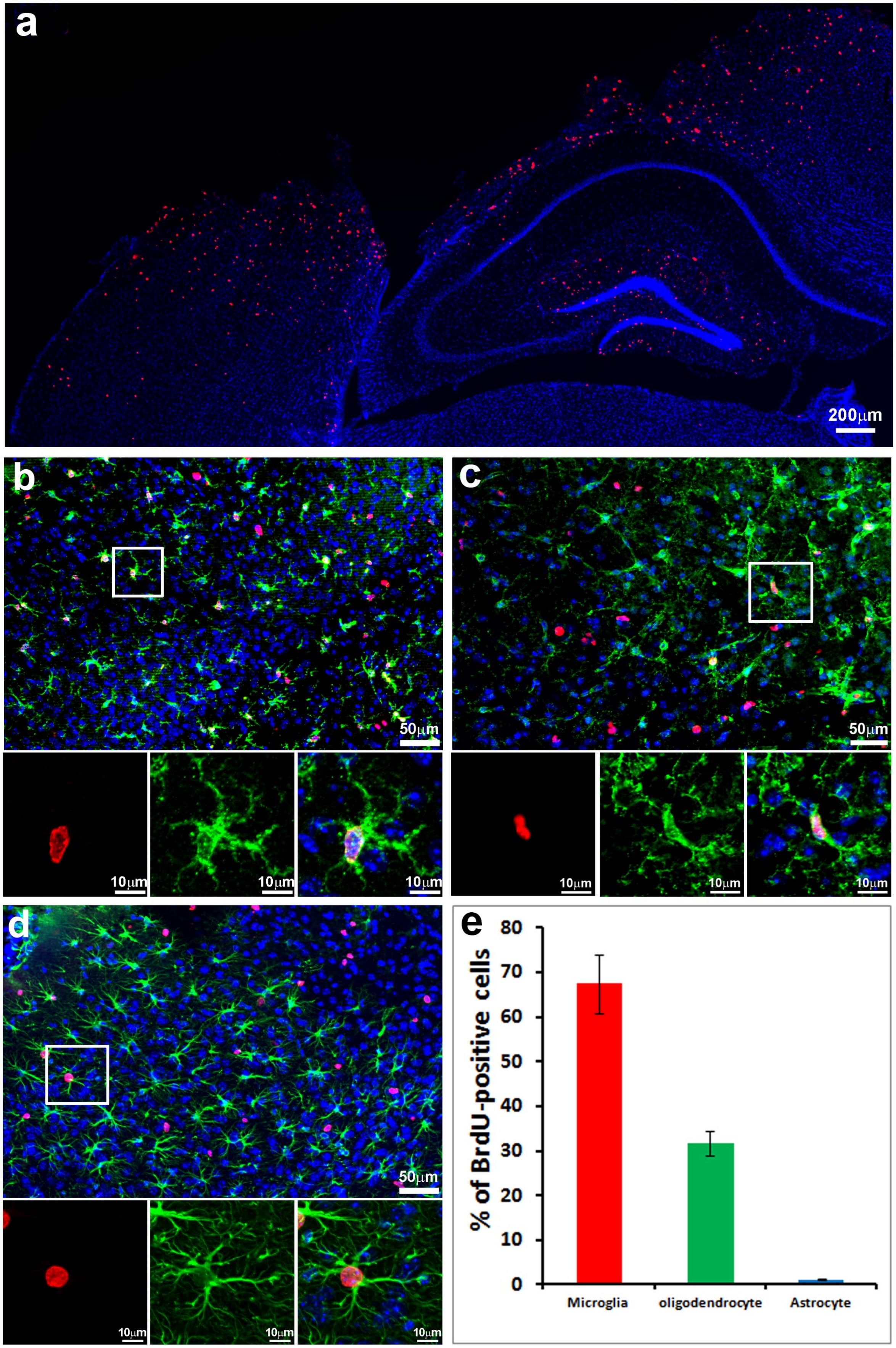

Supplemental figure 3

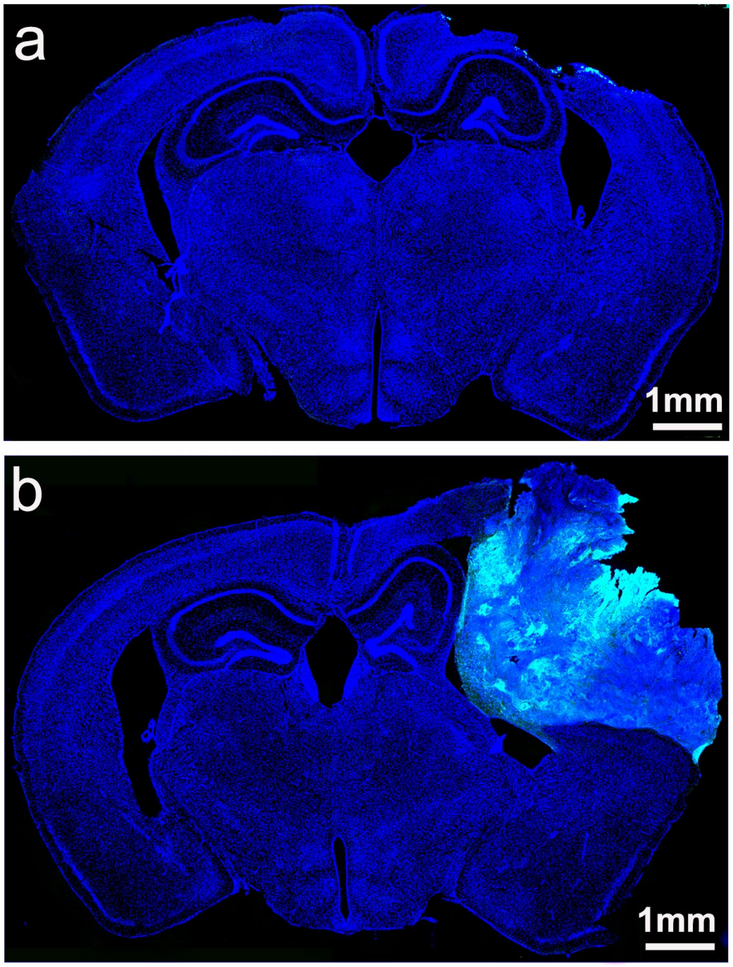

Supplemental figure 4

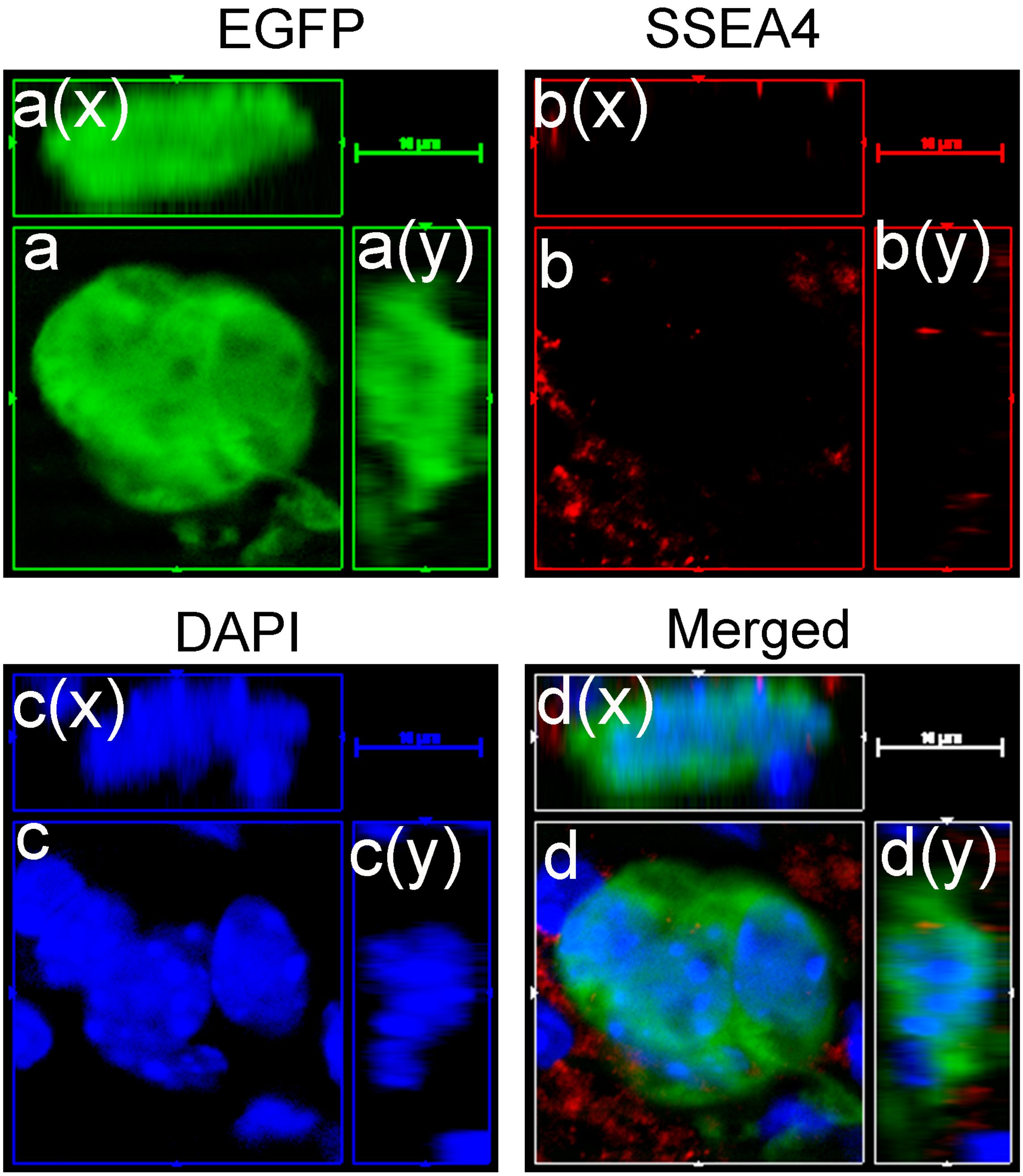

Supplemental figure 5

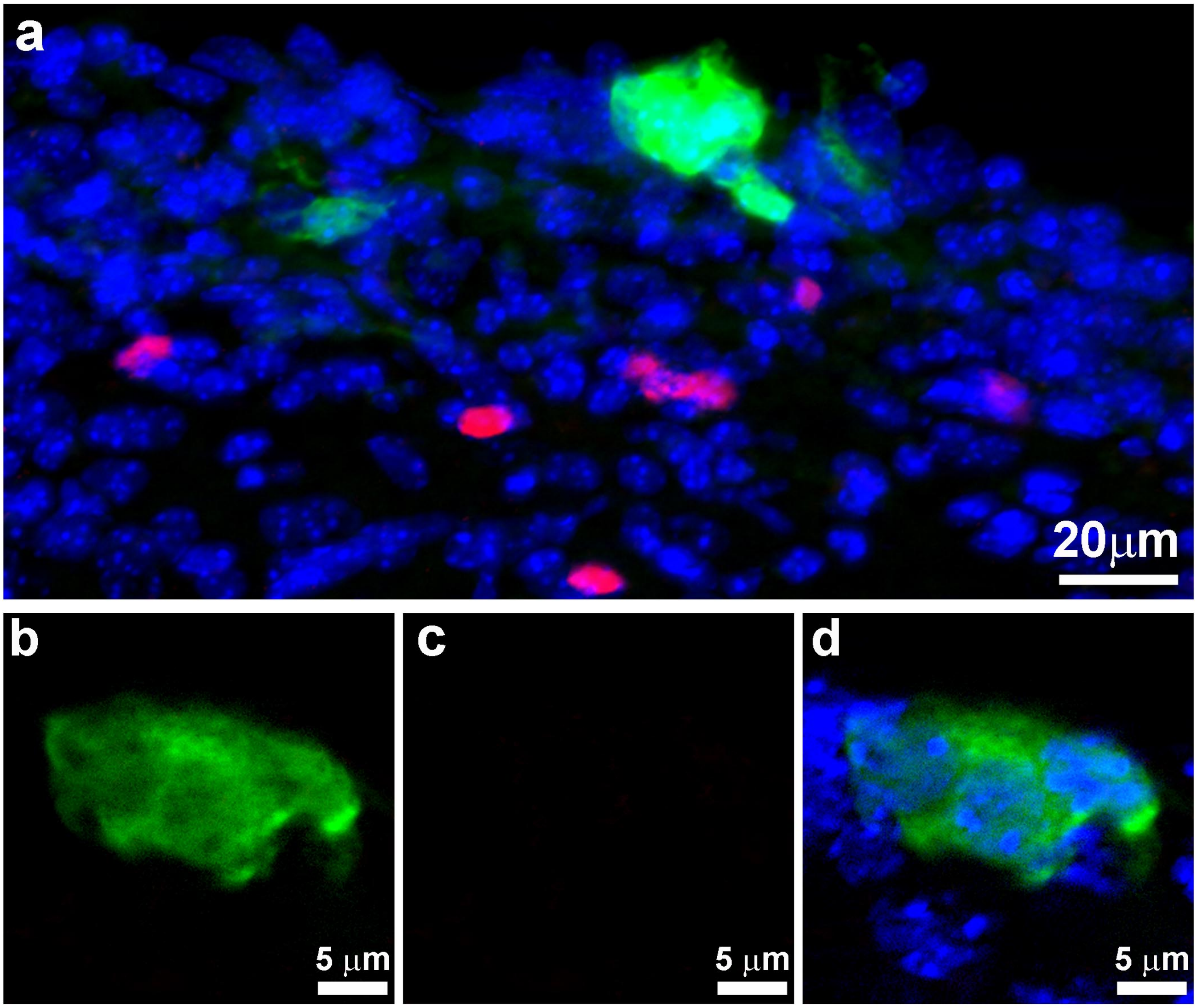

Supplemental figure 6

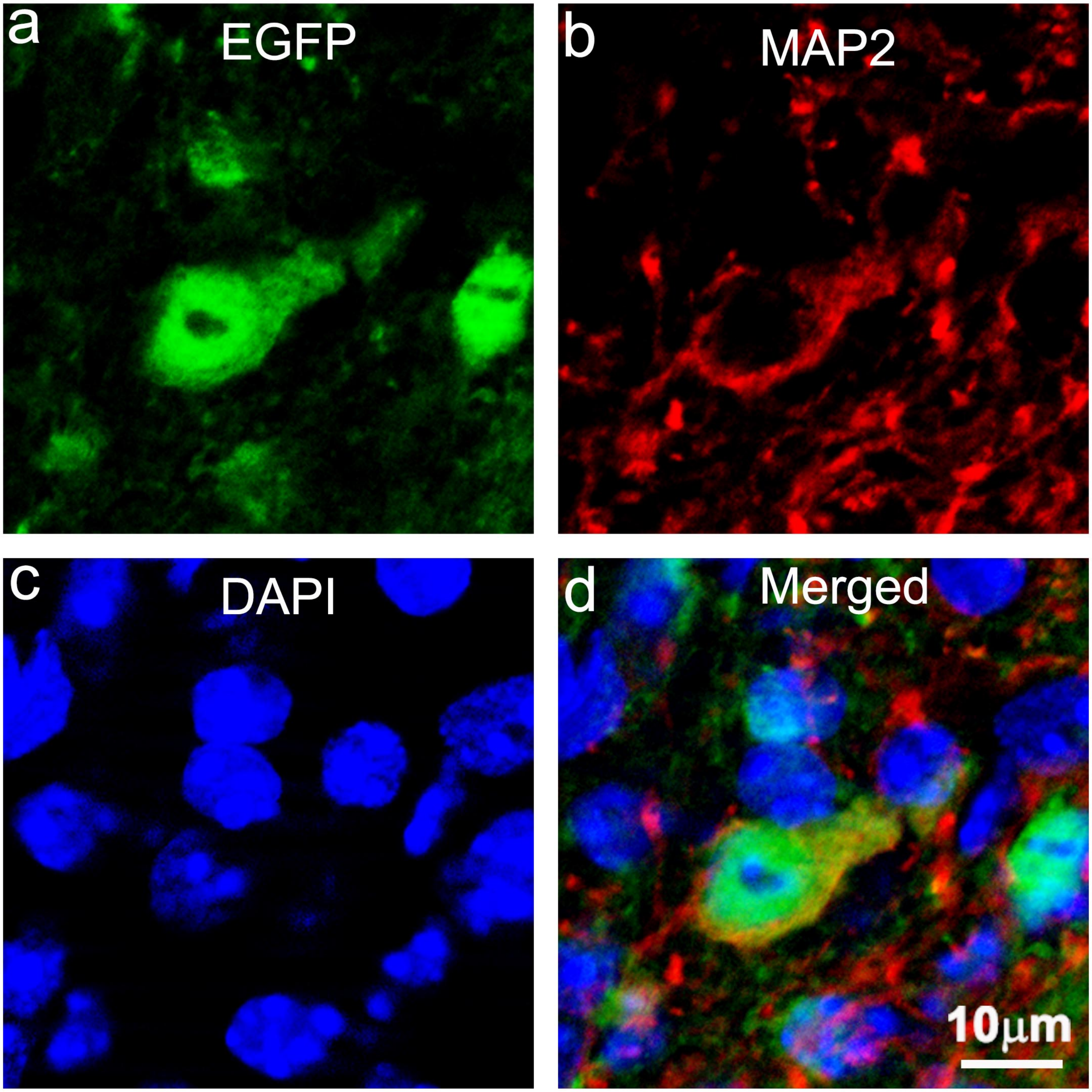

Supplemental Figure 7

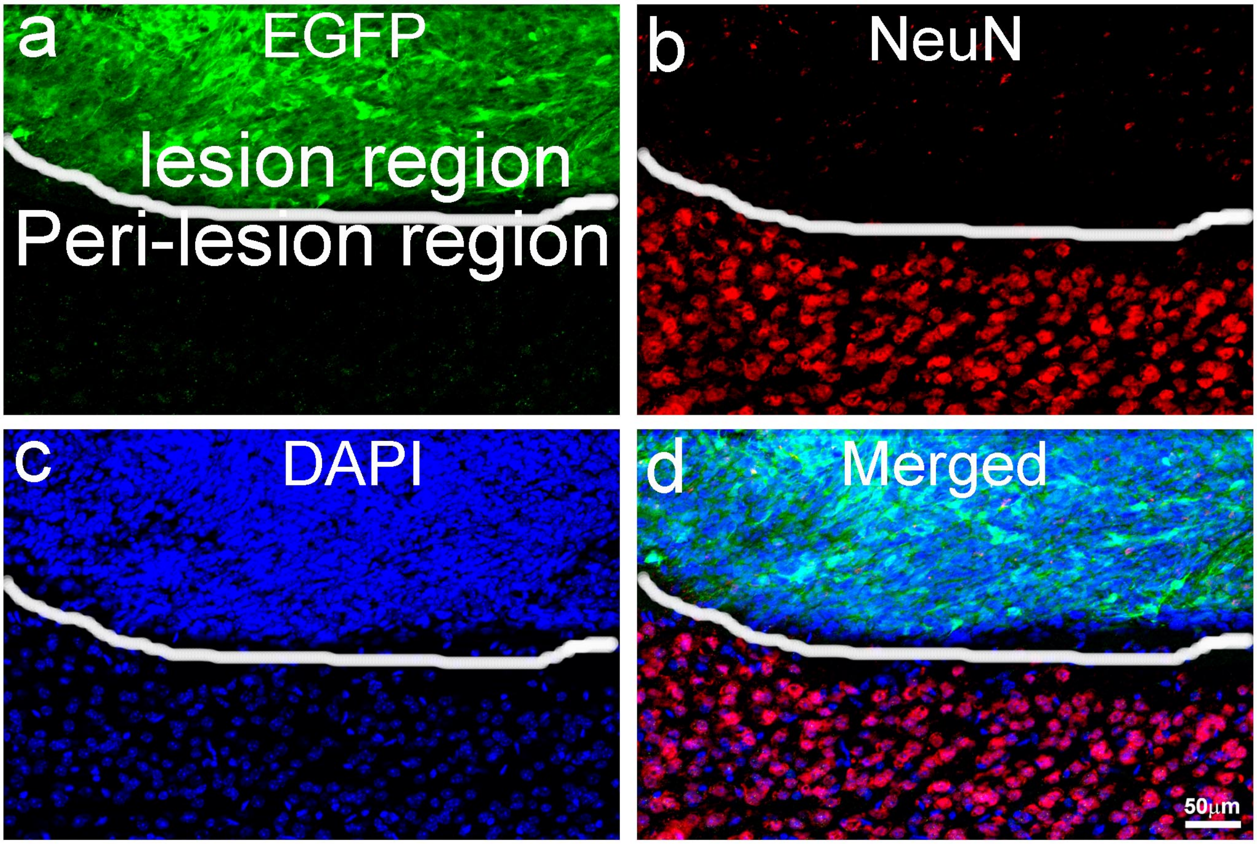

Supplemental figure 8

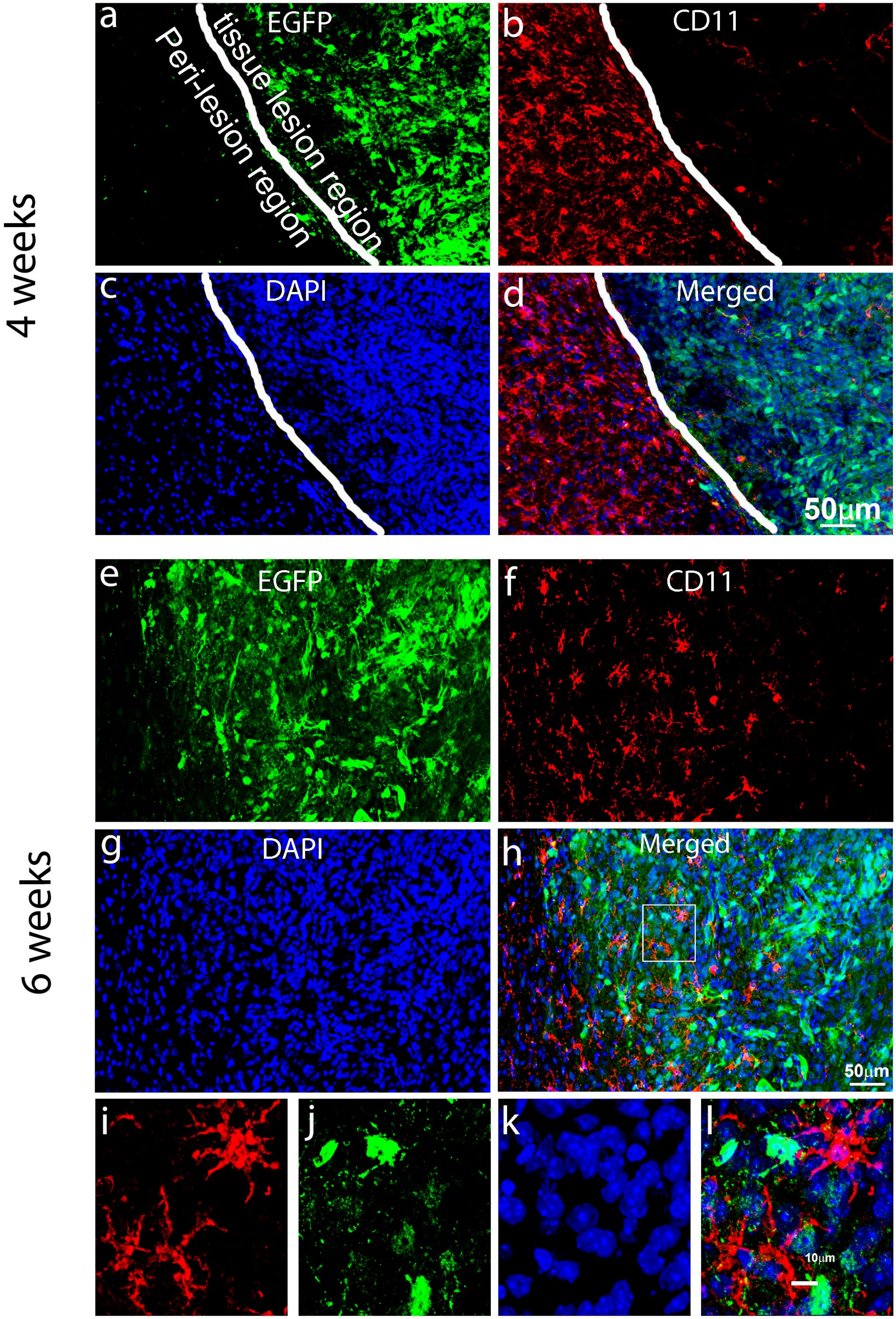

Supplemenal figure 9

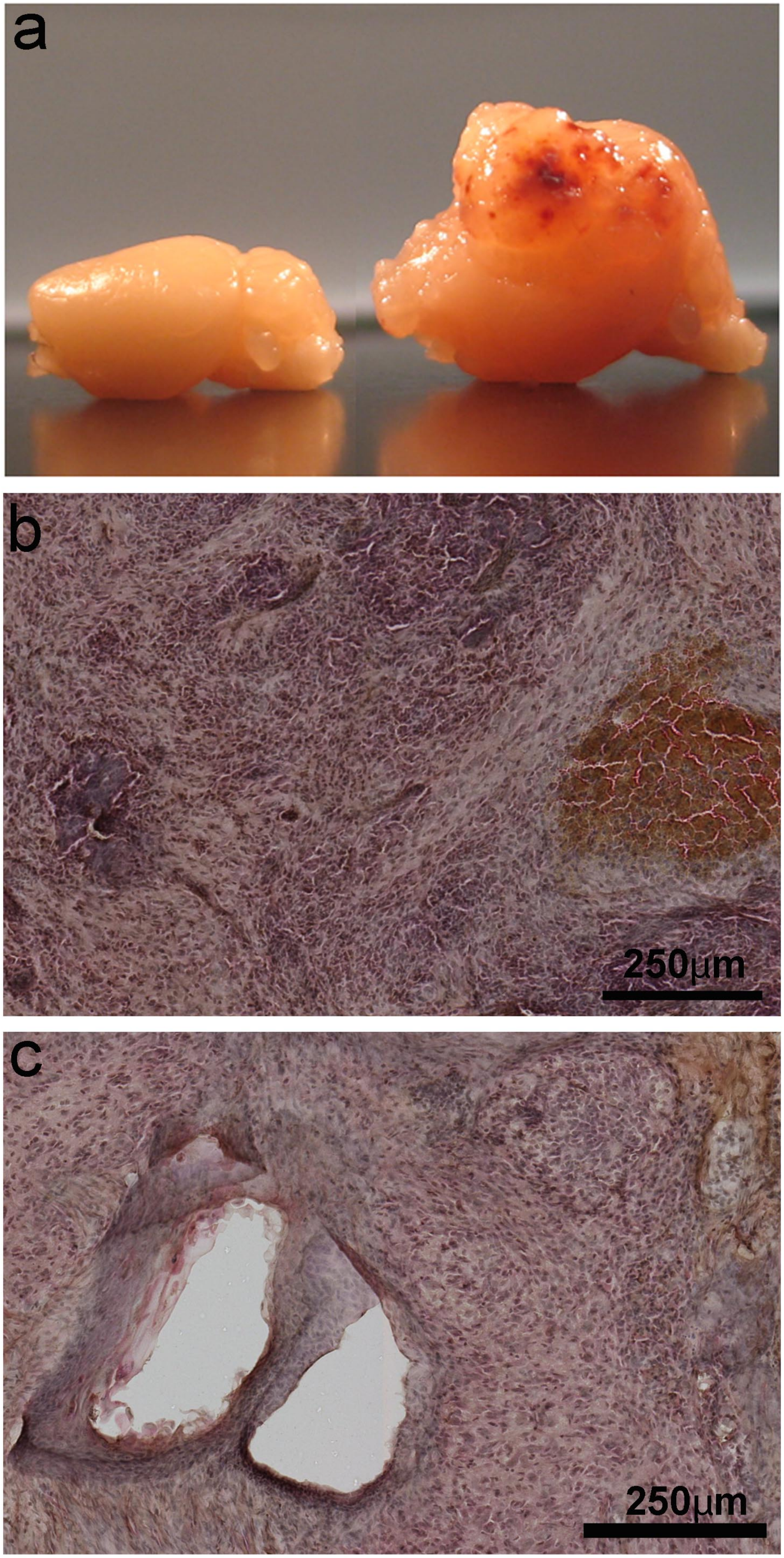

Supplemental figure legends

Supplemental figure 1. Retroviral dual cassette vectors co-expressing four human transcriptional factors (hOct4, hKLF4, hSOX2 and hcMYC, hOKSM) in combination with enhanced green fluorescent protein (EGFP).

a. Expression vector used for generating retrovirus contained 4 hOKSM factors, which can reprogram somatic cells into induced pluripotent stem cells and tagged by EGFP. b. Retrovirus infected proliferating cell and express EGFP (green) in vitro. The titration is about $1.6x10^{7}$ pfu.

Supplemental figure 2. Glial reactivation in the cortex following moderate traumatic brain injury (TBI).

a. Immunostaining with antibody against bromo-2'-deoxyuridine (BrdU) (red) to detect proliferating cells in the cortex 3 days after moderate traumatic brain injury (TBI). b. Majority of proliferating cells was Iba1-positive reactive microglia (green). c. Some proliferating cells expressed oligodendrocyte precursor marker NG2 (green). d. Very few proliferating cells were double-labeled by astrocyte marker-GFAP (green). e. Quantification data showed the ratio of different types of proliferating cells.

Supplemental figure 3. Reprogrammed cells fill the tissue-lesion cavity induced by traumatic brain injury (TBI).

a. TBI-injured brain received injection of controlled retroviruses expressing EGFP. b. TBI-injured brain received injection of retroviruses expressing defined transcriptional factors and EGFP. Green is EGFP, while blue is DAPI staining.

Supplemental figure 4. SSEA4 was not detectable in the cells expressing hOKSM-EGFP at 2 weeks after traumatic brain injury (TBI).

a. Three-dimensional images of cell cluster expressing hOKSM-EGFP 2 weeks after TBI. b. Three-dimensional image to show that the cell cluster did not express SSEA4 at 2 weeks after injury. c. 4′,6-diamidino-2-phenylindole (DAPI) staining to exhibit nuclei. d. Merged image of a-c.

Supplemental figure 5. SOX2 was not detectable in the cells expressing hOKSM-EGFP at 2 weeks after traumatic brain injury (TBI).

a. The hOKSM-EGFP-positive cell (green) clusters at the peri-lesion region did not express Sox2 at 2 weeks after TBI; instead, the Sox2 was expressed in cells that did not express EGFP, likely reactive glia, at the peri-lesion region of the injured cortex at 2 weeks after TBI. b-d, Enlarged images showed EGFP-positive cell aggregates without Sox2 expression 2 weeks after TBI.

Supplemental figure 6. Reprogrammed cells developed into Map2-expressing mature neurons at 6 weeks after traumatic brain injury (TBI).

a. The hOKSM-EGFP-expressing cells (green) in the TBI-injured cortex 6 weeks after reprogramming. b. The hOKSM-EGFP expressing cells differentiated into mature neurons expressing Map2 (red), a marker for mature neurons, in the injured cortex. c. DAPI staining. d. Merged image of a-c.

Supplemental figure 7. Reprogramed cells did not express NeuN at 4 weeks after traumatic brain injury (TBI). a. Tissue lesion cavity in the cortex following TBI was filled with reprogramming cells with EGFP expression (green). White line indicates the boundary between the tissue lesion cavity and peri-lesion region in the cortex at 4 weeks

after moderate TBI. b. Spared endogenous mature neurons expressing NeuN (red) located at the peri-lesion region. c. DAPI staining to exhibit nuclei in the cortex. d. Merged image of a-c.

Supplemental figure 8. Reprogramed cells did not differentiate into microglia.
a, e. Tissue lesion cavity in the cortex following traumatic brain injury (TBI) was filled with reprogramming cells with EGFP expression (green) at 4 or 6 weeks after moderate TBI. White line indicates the boundary between the tissue lesion cavity and peri-lesion region in the cortex. b, f. CD-11b positive microglia at the peri-lesion region in the cortex at 4 or 6 weeks after TBI. c, g. DAPI staining to exhibit nuclei in the cortex. d, h. Merged image of a-c or e-g. i-l. Enlarged images to show cells express either CD11b or EGFP within the white box in the panel h. The EGFP-positive cells did not express CD11b.

Supplemental figure 9. Reprogrammed cells developed into teratoma in the cortex.
a. Brain of control without reprogramming (left) or brain with reprogramming (right) at 6 weeks after TBI. b and c. H&E staining of the brain region with reprogrammed cells.
